# Alterations of Regional Cerebral Blood Flow in Tinnitus Patients as Assessed Using Single-Photon Emission Computed Tomography

**DOI:** 10.1371/journal.pone.0137291

**Published:** 2015-09-02

**Authors:** Takashi Ueyama, Tomohiro Donishi, Satoshi Ukai, Yuta Yamamoto, Takuya Ishida, Shunji Tamagawa, Muneki Hotomi, Kazuhiro Shinosaki, Noboru Yamanaka, Yoshiki Kaneoke

**Affiliations:** 1 Department of Anatomy and Cell Biology, Graduate School of Wakayama Medical University, Wakayama, Japan; 2 Department of System Neurophysiology, Graduate School of Wakayama Medical University, Wakayama, Japan; 3 Department of Neuropsychiatry, Graduate School of Wakayama Medical University, Wakayama, Japan; 4 Department of Otolaryngology-Head and Neck Surgery, Graduate School of Wakayama Medical University, Wakayama, Japan; University Zurich, SWITZERLAND

## Abstract

Tinnitus is the perception of phantom sound without an external auditory stimulus. Using neuroimaging techniques, such as positron emission tomography, electroencephalography, magnetoencephalography, and functional magnetic resonance imaging (fMRI), many studies have demonstrated that abnormal functions of the central nervous system are closely associated with tinnitus. In our previous research, we reported using resting-state fMRI that several brain regions, including the rectus gyrus, cingulate gyrus, thalamus, hippocampus, caudate, inferior temporal gyrus, cerebellar hemisphere, and medial superior frontal gyrus, were associated with tinnitus distress and loudness. To reconfirm these results and probe target regions for repetitive transcranial magnetic stimulation (rTMS), we investigated the regional cerebral blood flow (rCBF) between younger tinnitus patients (<60 years old) and the age-matched controls using single-photon emission computed tomography and easy Z-score imaging system. Compared with that of controls, the rCBF of tinnitus patients was significantly lower in the bilateral medial superior frontal gyri, left middle occipital gyrus and significantly higher in the bilateral cerebellar hemispheres and vermis, bilateral middle temporal gyri, right fusiform gyrus. No clear differences were observed between tinnitus patients with normal and impaired hearing. Regardless of the assessment modality, similar brain regions were identified as characteristic in tinnitus patients. These regions are potentially involved in the pathophysiology of chronic subjective tinnitus.

## Introduction

Tinnitus is the perception of phantom sound or noise in the absence of a real external auditory stimulus [1–4]. With a few exceptions, tinnitus is a highly subjective symptom that only patients can report. The prevalence of tinnitus is high (10%–15% among Western [5] and Japanese adults [6]). This condition has a substantial effect on community healthcare economics, as many patients need medical or psychiatric management from a range of specialists, including otorhinolaryngologists and practitioners of various complementary and alternative medicines [1–4]. However, effective and evidence-based treatments for tinnitus are limited [3, 4].

The etiology of tinnitus is debated between peripheral pathology, including cochlear and auditory nervous damage vs. dysfunction of the central nervous system (CNS) [7]. Recent improvements in neuroimaging techniques, such as positron emission tomography (PET), electroencephalography, magnetoencephalography, and functional magnetic resonance imaging (fMRI), indicate involvement of CNS in the pathophysiology of tinnitus [8–11]. These imaging investigations have demonstrated that brain regions considered to be irrelevant to the auditory system, such as the anterior cingulate gyrus, anterior insula, amygdala, hippocampus, and parahippocampal region, are deeply involved in the pathogenesis of subjective tinnitus. Using resting-state fMRI, we observed that non-auditory brain regions, such as the rectus gyrus, cingulate gyrus, thalamus, hippocampus, caudate, inferior temporal gyrus, cerebellar hemisphere, and medial superior frontal gyrus, were associated with tinnitus-related symptoms, such as distress, depression, and loudness [12].

Single-photon emission computed tomography (SPECT) is a functional neuroimaging method that can be used with 99 m Tc-ethylcysteinate dimer (99 m Tc-ECD) as a tracer to estimate regional cerebral blood flow (rCBF) [13]. Several studies have shown altered rCBF in tinnitus patients [14–19]. However, these studies were limited due to low numbers of control cases, lack of quantification, and ambiguous anatomical orientation. A recent study by Laureano MR et al. [20] is the first reliable SPECT study with sufficient number of control cases (n = 17), sound statistical methods, and precise anatomical orientation using MRIcron [21]. They demonstrated that the rCBF in the left parahippocampus was significantly increased in tinnitus with normal hearing.

The easy Z-score imaging system (e-ZIS) for SPECT was developed by Matsuda H et al. [22–27] and is commercially available (Fuji Film, Tokyo, Japan). This system has the following advantages: (1) precise anatomical standardization using statistical parameter mapping, (2) large number of subjects (n = 138) from an age-matched control database, including 20–39 year olds (n = 28), 40–59 year olds (n = 30), 60–69 year olds (n = 40), and 70–86 year olds (n = 40), (3) ability to perform brain phantom image adjustments and standardization between different SPECT systems, and (4) statistical analytical functions using a built-in database. Moreover, the combination of SPECT and e-ZIS can discriminate very early Alzheimer’s disease from controls [25–27].

This study intended to reconfirm the results of our previous resting-state fMRI study using a different modality and method. We used SPECT and e-ZIS to compare rCBF between young/middle-aged tinnitus patients and controls. This study also intended to identify therapeutic target regions for repetitive transcranial magnetic stimulation (rTMS). Previous studies have used the primary auditory cortex as the target of rTMS [28]. Our attempt to treat tinnitus using rTMS on the primary auditory cortex was unsuccessful [29]. Further investigation to identify a different target for rTMS is needed.

## Materials and Methods

### Subjects

Seventeen young and middle-aged subjects (25–59 years old) were re-enrolled in this study with written consent. The subjects were outpatients (August 2013–May 2015) at the clinic of the Department of Otolaryngology-Head and Neck Surgery at Wakayama Medical University Hospital. This study was conducted according to the Declarations of Helsinki with the approval of the Wakayama Medical University Ethics Committee (No. 1265). The subjects (10 males and 7 females) did not have a history of seizures or a suspected diagnosis of organic brain damage, brain tumors, or psychiatric diseases (including major depressive disorder) prior to the onset of tinnitus. No patients received medication with antianxiety, antidepressant, anti-epileptic, or other psychotropic drugs. Tinnitus-related distress was assessed using the standard tinnitus handicap inventory (THI) [30]. Statistical analysis of patient profiles was conducted using the Student’s t-test and chi-square test on MATLAB software (Mathworks, Natick, MA, USA).

### Audiological Examination

Expert otorhinolaryngologists made the determination of normal middle ear status. Hearing levels of subjects were determined using a pure-tone audiometer (AA-78, RION, Tokyo, Japan). Subjects were presented with pure tones ranging from 250 Hz to 12 kHz to each ear until the detection threshold was reached. The number of subjects with normal hearing (hearing level in any frequency <30 dB) was 6, while that with mild to severe hearing loss was 11.

### SPECT and CBF analysis

Relative CBF was estimated using the 99 mTc-ECD-SPECT as previously described [22–27, 29]. All subjects received an intravenous injection of 600–640 MBq 99 mTc-ECD (Neurolite injection Daiichi, FUJI FILM, Tokyo, Japan) while in the supine position with eyes closed in a dark and quiet room. The background noise level in the room was approximately 50 dB. The SPECT images were acquired based on previously reported protocols [29]. Briefly, data were collected using a double gamma camera (Millennium VG; GE, Yokogawa, Japan) with the acquisition parameters as described [29].

### e-ZIS

Methods used were based on previously-reported protocols [26]. We used the e-ZIS program, version 4 (FUJI FILM, Tokyo, Japan) for analysis of brain perfusion SPECT images to discriminate between young/middle-aged tinnitus patients and age-matched healthy controls grouped by age as follows: 20–39 years (n = 28) and 40–59 years (n = 30). A positive Z-score indicates lower rCBF than that of the control database. Z-scores were 3-dimensionally mapped using MRIcron [21]. Correction for multiple comparisons was based on a previously reported procedure [12].

The Z-scores at the peak voxel in several brain regions were compared between subjects with normal and impaired hearing using Student’s t-test with MATLAB software (Mathworks, Natick, MA).

## Results

Patients were divided into two groups based on their hearing acuity as follows: normal hearing, hearing level in any frequency <30 dB; impaired hearing, mild to severe hearing loss (Table 1).

**Table 1:** The profiles of patients.

		Normal hearing	Impaired hearing	P
Sex	Male	5	5	0.13
	Female	1	6	
Age (Mean ± SD)		44.7 ± 10.0	50.6 ± 9.8	0.25
Mean hearing level	L	17.5 ± 5.7	21.2 ± 5.2	0.19
	R	15.5 ± 3.9	25.6 ± 17.7	0.19
THI		54.7 ± 23.1	51.9 ± 27.9	0.84
Laterality	L	3	3	0.78
	R	1	2	
	Bilateral	1	4	
	Brain	1	2	

Three subjects did not identify the laterality of their tinnitus, reporting “my tinnitus seems to be audible from my brain not from my ears.” There were no significant differences in sex, THI, ages, or mean hearing levels between patients with normal and impaired hearing.

**Fig 1:**
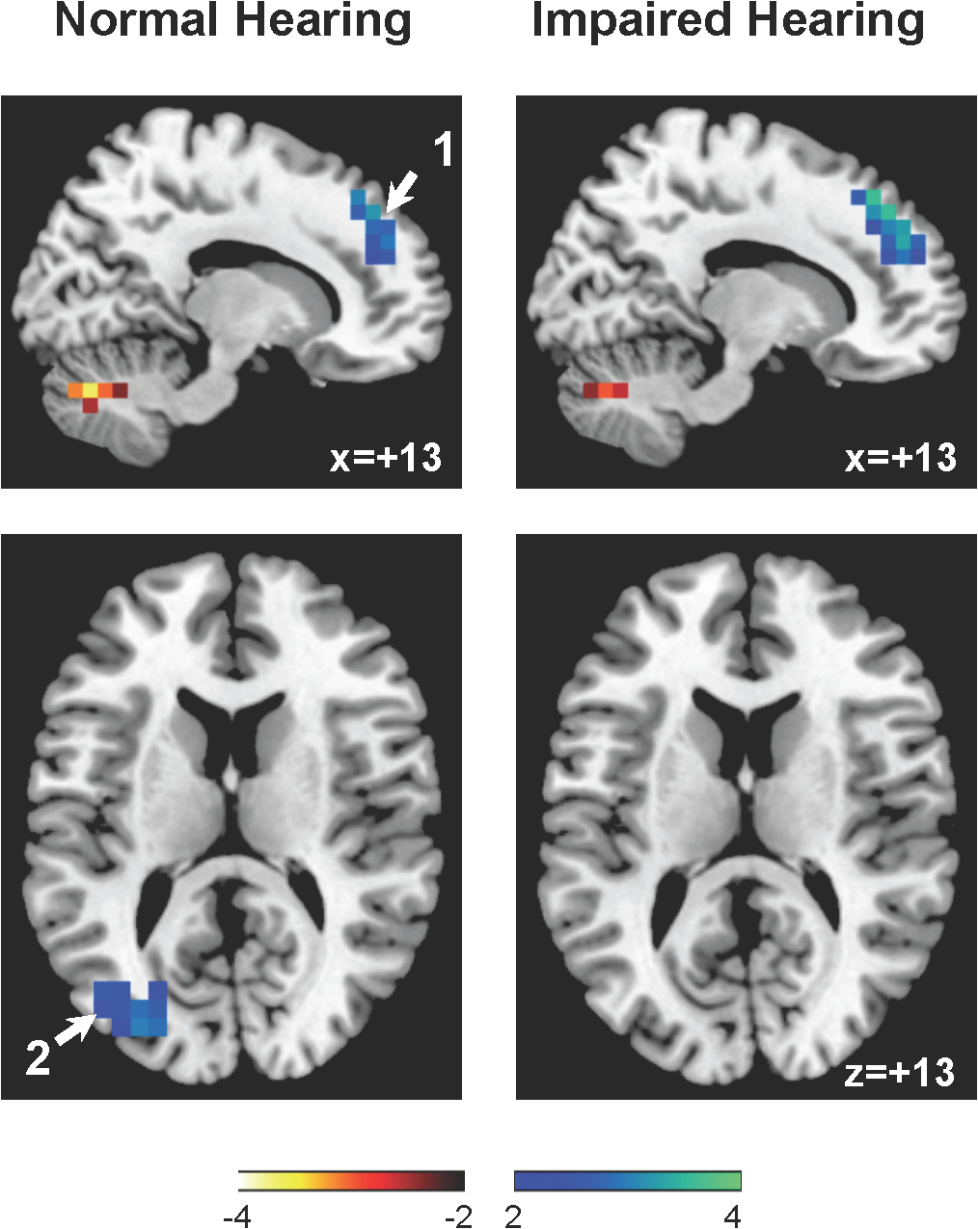
Significant decreases in rCBF (colored in blue). 1: the right medial superior frontal gyrus and 2: the left middle occipital gyrus.

**Fig 2:**
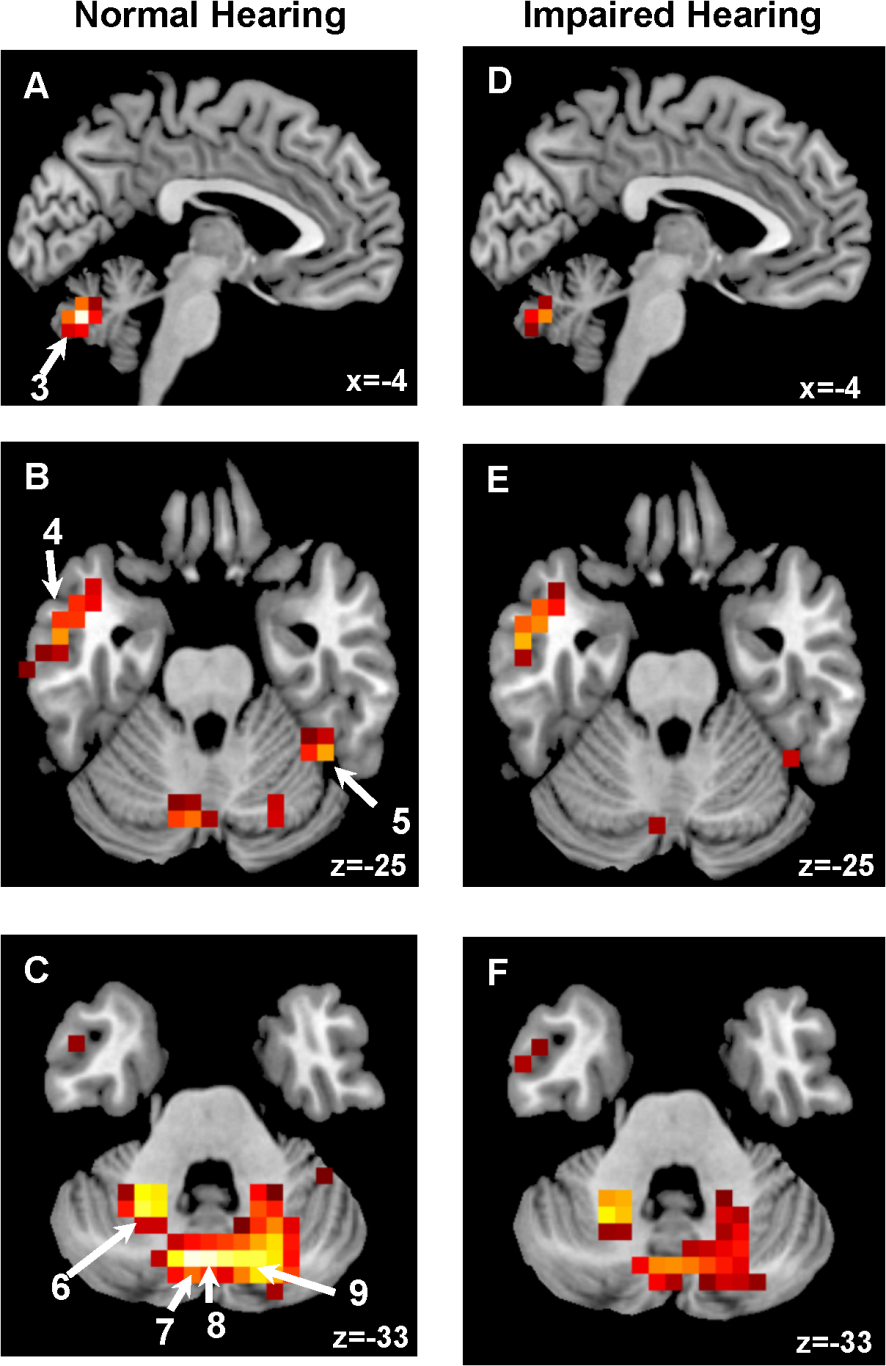
Significant increases in rCBF (Colored in red). 3: the left cerebellum Crus 2, 4: left middle temporal gyrus, 5: right fusiform gyrus, 6: left cerebellum Crus 6, 7: left cerebellum Crus 2, 8: vermis 7, and 9: right cerebellum Crus 2, respectively.

The distribution pattern of Z-scores in Fig 1 (Significant hypoperfusion) and Fig 2 (Significant hyperperfusion). Significant decreases in rCBF were observed in the bilateral medial superior frontal gyri and left middle occipital gyrus in patients with normal and with impaired hearing. Significant increases in rCBF were detected in the bilateral middle temporal gyri, right fusiform gyrus, and bilateral cerebellar hemispheres and vermis. Table 2 compares peak voxel Z-scores in several brain regions between tinnitus patient with normal and impaired hearing; no clear differences were observed between those with normal and impaired hearing.

**Table 2:** Z-score at the peak voxel in several brain regions.

Region	MNI			BA	Normal hearing	Impaired hearing	P
	X	Y	Z		Z-score (mean ± SD)		
right medial superior frontal gyrus	11	44	40	9	3.19 ± 0.57	3.58 ± 0.72	0.27
left middle temporal gyrus	−53	−6	−25	48	−3.18 ± 0.88	−3.28 ± 0.64	0.80
right fusiform gyrus	37	−47	−19	37	−3.39 ± 0.50	−2.87 ± 0.62	0.10
left cerebellum Crus2	−4	−72	−33		−3.96 ± 1.31	−3.15 ± 1.47	0.28
right cerebellum Crus1	17	−72	−33		−3.64 ± 1.14	−2.82 ± 0.96	0.13
Vermis 7	−1	−73	−33		−3.91 ± 1.31	−3.17 ± 1.66	0.36
left cerebellum 6	−25	−56	−33		−3.63 ± 1.21	−3.48 ± 0.57	0.72
left middle occipital gyrus	−30	−82	16	19	3.42 ± 0.77	2.72 ± 0.74	0.09

The Z-scores in the primary auditory cortex (Heschl gyrus) were within ±1 in all patients examined.

## Discussion

Regardless of hearing acuity, tinnitus patients exhibited alterations in rCBF in the bilateral medial superior frontal gyri, bilateral cerebellar hemispheres, cerebellar vermis, bilateral middle temporal gyri, bilateral fusiform gyri, and bilateral middle occipital guri. These regions were almost identical to those associated with tinnitus symptoms as revealed by resting-state fMRI [12].

The commercially provided control database does not include audiological information; thus, might include subjects, especially in aged subjects with mild hearing loss and tinnitus. However, few subjects in the young and middle-aged control group are likely to have hearing loss and tinnitus. Therefore, we examined differences in rCBF between young and middle-aged, medication-free patients compared with age-matched controls.

Significant hypoperfusion was observed in the bilateral medial superior frontal gyrus, where a significant negative correlation was observed between the regional global connectivity (based on functional connectivity as measured by resting-state fMRI) and tinnitus loudness [12]. This negative correlation suggests that the medial superior frontal gyrus does not coordinate with other brain regions in patients with more severe tinnitus. The medial superior frontal and posterior cingulate gyri are parts of the default mode network (DMN) consisting of brain regions that become activated when an individual is not focused on external stimuli [31]. These observations suggest that DMN function is impaired in tinnitus. A negative correlation between *rGCa* and tinnitus loudness was also detected in the posterior cingulate gyrus, while hypoperfusion was not detected in any tinnitus patients. As hypoperfusion in the posterior cingulate gyrus is diagnostically significant in very early Alzheimer’s disease [25–27], our tinnitus patients were free of this condition. Several reports have described the involvement of the medial superior frontal gyrus in normal or abnormal auditory functions [32–34]. These observations suggest that the medial superior frontal gyrus may be involved in the pathophysiology of tinnitus and hearing. However, the present evidence is insufficient to draw conclsions.

Hyperperfusion was observed in the bilateral middle temporal gyri and right fusiform gyrus. This result was similar to a recent report that the left parahippocampus was significantly hyperperfused in tinnitus with normal hearing [20]. In our study, other regions can be demonstrated in subjects with both normal and impaired hearing. Our previous study using a resting-state fMRI has also demonstrated a significant positive correlation between *rGCa* and tinnitus distress in the right inferior temporal gyrus. A significant positive correlation between *rGCa* and tinnitus loudness was observed in the bilateral hippocampus. These studies emphasize the contribution of the lower temporal lobes, including the amygdala, hippocampus, parahippocampus, and fusiform gyrus, to tinnitus perception.

Remarkable hyperperfusion was also observed in both cerebellar hemispheres and cerebellar vermis. In the right cerebellar hemisphere, a significant positive correlation was observed between *rGCa* and tinnitus distress [12]. Several lines of study have indicated that the cerebellum is involved in the auditory system [35–37]. The lateral cerebellum and the primary auditory cortex were both activated by exposure to a 4.0-kHz sound [35]. The posterolateral portion of both cerebellar hemispheres in crus II, lateral to the paravermian region, was activated by auditory stimulation at 40 Hz [36, 37]. Functional connectivity between the auditory cortex and other cortices, including the cerebellum, was observed to be higher in tinnitus patients [38, 39]. A recent experimental animal study has demonstrated that the paraflocculus (PFL) of the cerebellum is a generator of tinnitus [40]. Surgical ablation or inactivation with lidocaine in the PFL eliminated acoustic trauma-induced tinnitus in rats. The cerebellum may be involved in the depression-like behavior of rats caused by decreased growth hormone gene expression [41]. Together, our results and these recent observations strongly suggest the contribution of the cerebellum to auditory function and emotion as well as tinnitus symptoms. However, the underlying mechanism cannot be determined from these limited observations.

Many studies have indicated the involvement of the primary auditory cortex (Heschl gyrus) in tinnitus. Elevated metabolic activity in the left primary auditory cortex has been reported [42]. This hyperactivity in the left primary auditory cortex is believed to be caused by repetitive transcranial magnetic stimulation in tinnitus [28]. In contrast, this study, together with our previous report [12] and that of Laureano et al. [20], detected no changes in rCBF or *rGCa* in the primary auditory cortex. A recent study using PET also showed that the activity in the primary auditory cortex did not differ between tinnitus patients and controls [43]. These recent studies suggest minimal involvement of the primary auditory cortex in the pathology of tinnitus.

### Limitations

The commercially-provided control database might include patients with mild hearing loss and/or tinnitus, although these controls were healthy and neurologically normal. In this study, we compared young (age <40 years) and middle-aged (age 40 ~59), medication-free subjects with those of the normal control datebase (20–39 years, n = 28 and 40–59 years, n = 30) provided by Fuji Film because it is unlikely that these control subjects had audiological disorders. However, it should be careful to conclude that these regions are specific to tinnitus itself

Whether the altered rCBF in these regions is causative of tinnitus or secondary or compensatory changes in response to tinnitus remain unclear. Changes in rCBF may be affected by age, for which we corrected using a corresponding age-matched control group. Ambient noises during image acquisition of SPECT may affect the resting-state rCBF depending on the hearing acuity of patients. However, hearing acuity did not influence the change in rCBF in tinnitus. The laterality and duration of tinnitus could not be taken into consideration in this study. These factors may affect the laterality and degree of rCBF in tinnitus.

## Conclusions

Regardless of hearing acuity, rCBF in tinnitus patients was lower in DMN regions, such as the bilateral medial superior frontal gyri, and higher in memory and emotional networks, such as the bilateral lower temporal lobe, compared with that in normal controls. Hyperperfusion was also observed in the bilateral cerebellar hemispheres and vermis. These regions were almost identical to those exhibiting an association between the *rGCa* and tinnitus symptoms. In addition, these regions may be possible targets for tinnitus treatment.

## Supporting Information

S1 Data Includes the Z-scores (e-ZIS) in brain regions of each subject as NIFTI files (*.hdr and *.img). The name of each NIFTI file indicates patient number as shown in S1 List.
(ZIP)

S1 List Profile of corresponding subjects as EXCEL file (*.xlsx). (ZIP)
